# Duchenne muscular dystrophy caused by a frame-shift mutation in the acceptor splice site of intron 26

**DOI:** 10.1186/s12881-016-0318-y

**Published:** 2016-08-11

**Authors:** Mirella Meregalli, Simona Maciotta, Valentina Angeloni, Yvan Torrente

**Affiliations:** Department of Phatophysiology and Transplantation, Stem Cell Laboratory, Università degli Studi di Milano, Fondazione IRCCS Ca’ Granda Ospedale Maggiore Policlinico di Milano, Centro Dino Ferrari, via F. Sforza 35, 20122 Milan, Italy

**Keywords:** DMD, Dystrophin gene, Frame-shift mutation

## Abstract

**Background:**

The dystrophin gene is the one of the largest described in human beings and mutations associated to this gene are responsible for Duchenne or Becker muscular dystrophies.

**Case Presentation:**

Here we describe a nucleotide substitution in the acceptor splice site of intron 26 (c.3604-1G > C) carried by a 6-year-old boy who presented with a history of progressive proximal muscle weakness and elevated serum creatine kinase levels. RNA analysis showed that the first two nucleotides of the mutated intron 26 (AC) were not recognized by the splicing machinery and a new splicing site was created within exon 27, generating a premature stop codon and avoiding protein translation.

**Conclusions:**

The evaluation of the pathogenic effect of the mutation by mRNA analysis will be useful in the optics of an antisense oligonucleotides (AON)-based therapy.

## Background

Muscular dystrophies include more than 30 inherited disorders causing muscle to weaken and wither away, and Duchenne Muscular Dystrophy (DMD) represents the most severe form of this family of disorders. It is an X-linked disease, affecting 1 in 5000 male births and is caused by the absence of the protein dystrophin in skeletal, cardiac and smooth muscle. Clinical symptoms manifest at about 3 years of age, and with the progressive muscle impairment patients are wheelchair bound by their early teen years and suffer cardiac/respiratory failure in their mid- to late twenties [1]. In particular, the incidence of cardiomyopathy in DMD increases with age. Around 25 % of patients have cardiomyopathy at 6 years of age and 59 % from 10 years of age, in older patients cardiac involvement is ubiquitous, as more than 90 % of young men over 18 years of age demonstrate evidence of cardiac dysfunction. No cure is nowadays available and current treatments include steroid administration and assisted ventilation [2].

Dystrophin is a cytoplasmatic protein located at the membrane of muscle fibres (sarcolemma) representing a connection between the extracellular matrix and the cytoplasmatic compartment of muscle fibres. For this reason mechanical stress like contraction during normal exercise impairs dystrophic muscle fibres that degenerate and, as time goes by, non-contractile fibrotic tissue replaces muscle tissue [1]. Dystrophin, other than having a mechanical role, takes part in several signalling pathways spanning from Ca^2+^ through Nitric Oxide (NO) synthesis and Ras/MAPK pathway, underling even more the relevancy of this protein in muscle physiology.

In DMD patients absence of dystrophin is caused by frameshift mutations in the *DMD* gene that give origin to premature stop codons and avoid protein translation. There are also mutations that maintain the open reading frame (ORF) of the *DMD* gene leading to a milder form of dystrophy called Becker Muscular Dystrophy (BMD). BMD subjects are in fact characterized by low levels of full-length dystrophin or carry in-frame mutations that allow the generation of shorter but functional forms of dystrophin. The onset of symptoms in BMD subjects is usually in late childhood or adolescence, and the course is slower and less predictable than that of DMD.

The *DMD* gene is one of the largest in the human genome (approximately 2.22 million base pairs, encoding 79 exons) and it is characterized by a frequency of mutation > 8 × 10^−5^ [2] (OMIM; 300376). 65 % of DMD patients harbour dystrophin gene deletions in a commonly mutated region including exons 45–55 with genomic breakpoints (ie, the endpoint of where the deletions actually occurs) lying within intron 44, and exons 2–19 with genomic breakpoints commonly found in introns 2 and 7 and alsoextending toward the downstream introns (OMIM; 300376) [3]. The clusters of these two hotspots represent the basis for the use of the multiplex PCR technique that, by the screening of only 19 exons, identifies about 98 % of all deletions [4–7]. Nevertheless, the multiplex PCR has been superseded in many laboratories by the convenience and commercial availability of multiplex ligation-dependent probe amplification (MLPA). MLPA is a quantitative assay of all exons of the *DMD* gene and the use of this technique strongly improves the mutation detection rate. Before MLPA was used as a routinely diagnostic tool, in fact, exon duplications within the *DMD* gene were estimated to be 5 % of all mutations responsible for DMD but the use of quantitative assay like MLPA raised this percentage to 10. The remaining ones are small insertions/deletions or point mutations and they are identified by direct sequencing of dystrophin gene. Point mutations also generate splicing defects in the donor/acceptor splice sites that cause exon skipping, intron retention or activation of cryptic splice sites within an exon or intron.

Here we describe a 6 year-old boy with a clinical history of progressive proximal muscle weakness who was diagnosed with DMD. Our major aim was to identify the mutation responsible for DMD and understand its pathogenic effect in order to confirm the clinical diagnosis with a molecular mechanism. Identifying the mutation also reduces the risk of recurrence of the disease in the family and minimize anxiety in the relatives of the patients. Moreover, understanding its pathogenic effect highlights the potential therapeutic approaches relevant for the patient and might help identifying the correlation between the genotype and the phenotype.

## Results and discussion

### Case report

The patient was diagnosed with DMD at 3 years of age on the basis of clinical presentation, elevated serum creatine kinase (CK) (26678 U/L), and negative dystrophin staining on immunohistochemistry. At the beginning he developed a sudden appearance of laterocervical tumefaction, not due to EVC, CMV and toxoplasm infections. Over the next year he developed troubles playing sports, climbing stairs and keeping up with peers. Early development was normal and he achieved above average academic performance. He has no affected brother and sister. On examination at 6 years of age cardiac and general exam were normal. Muscle bulk was normal with the exception of notable enlargement of calf muscles. A mildly increased lumbar lordosis was also present. Strength testing using the Medical Research Council (MRC) grading revealed weakness in the following distribution: femoral quadriceps 4 bilateral, ileopsoas 4- bilateral, tibialis anterior 4/5 bilateral, gastrocnemius 4/5 bilateral, tibialis posterior 4/5 bilateral. He utilized the Gowers’ manoeuvre to rise from the floor in 6–7 s. He was able to climb stairs using banister or a person, he had never been able to run as quickly as his peers and he showed an ambulation steppage gait. He did not require ambulatory aids but he showed limited endurance, he was idler and renouncer. Prolonged walking for greater than 30 min led to muscle discomfort in the calf muscles, and a brief period of rest was required to eliminate the discomfort as well as to alleviate fatigue. Laboratory studies revealed elevated alanine transaminase (ALT) at 397 U/L and aspartate transaminase (AST) at 190 U/L, elevated glucose level (121 mg/dl) and low iron level 56mcg/dl. Serum CK was 11375 U/L. Echocardiogram was normal with no evidence of myocardial hypertrophy.

### Immunohistochemistry

**Fig. 1:**
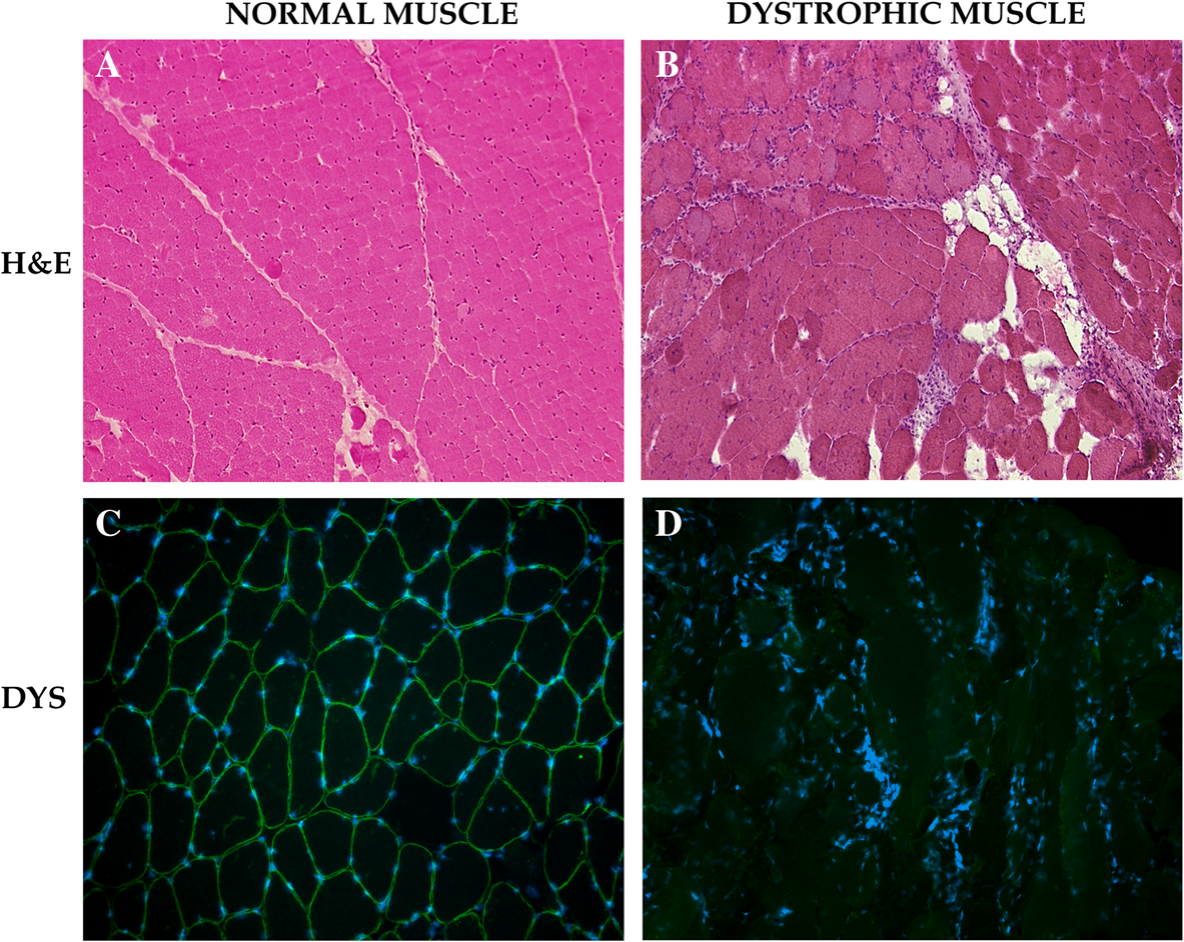
Histological analysis of control and DMD patient. Muscle sections were stained with Hematoxylin-Eosin (**a**-**b**). Normal muscle was compared to dystrophic one (**a**). The dystrophic muscle presented marked variability in fiber size, fiber splitting, scattered necrotic and regenerating fibers, and prominent endomysial and perimysial fibrosis (**b**). Muscle sections cut from normal (**c**) and dystrophic muscle were stained with dystrophin antibodies (**d**) in green. Nuclei were stained with DAPI

Muscle sections of the DMD patient and a control subject were stained with H&E. Respect muscle of control muscle (Fig. 1a), dystrophic muscle sections were characterized by fibres of variable cross sectional area (CSA), scattered necrotic and regenerating fibres and by prominent endomysial and perimysial fibrosis (Fig. 1b).

In order to confirm the absence of dystrophin protein in the patient, muscle sections cut from frozen biceps brachialis of the DMD patient and healthy subject were stained for dystrophin (Fig. 1c–d). This analysis evidenced the absence of dystrophin protein in muscle of DMD patient (Fig. 1d). Otherwise, control muscle presented a normal expression of dystrophin which is localized under the sarcolemma (Fig. 1c). Moreover, staining for sarcoglycans evidenced only a marked down-regulation of alpha-sarcoglycan in dystrophic patients (data not show).

### Mutational analysis

**Fig. 2:**
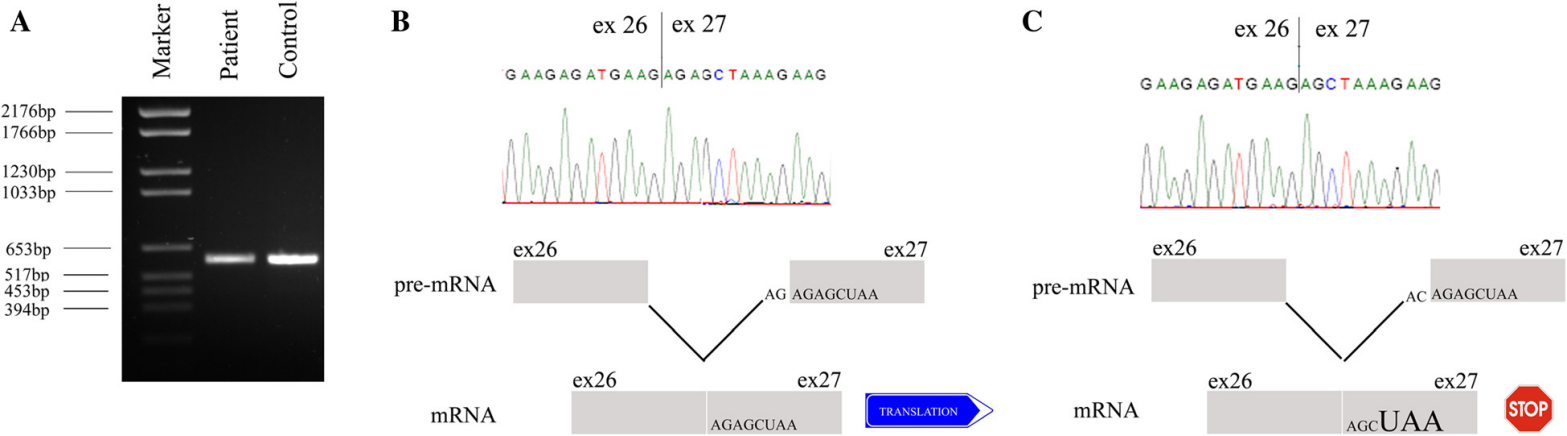
Functional analysis of DMD mutation. PCR analysis from control and DMD mRNA isolated form peripheral leukocytes was performed in order to understand if the mutation in the acceptor splice site of intron 26 (AG → AC) was responsible for uncorrect splicing and abnormal dystrophin transcript. Both samples control and DMD samples apparently produced a single band of the expected size (exons 25–29) (**a**). Direct sequence analyses confirmed that the band obtained by PCR corresponded to the full-length isoform of dystrophin for both control and DMD samples (**b**-**c**). Dystrophin mRNA of the DMD patient was found to miss the first two nucleotides (AG) of exon 27. The mechanism proposed is that in the presence of the mutated acceptor splice site of intron 26 (AC), the first two nucleotides of intron 27 are recognized as a new acceptor splice site due to sequence homology and are not included in the mRNA. The elimination of nucleotides AG from exon 27 generates a STOP codon in exon 27 that do not permit the production of a functional dystrophin (**c**)

Genomic DNA was isolated from peripheral leukocytes obtained from the DMD patient. Genomic DNA was analyzed by MLPA using a commercial kit (MRC Holland). Deletions or duplications of any of the 79 exons in the dystrophin gene were not detected. Next step was to scan the *DMD* gene for nucleotide changes. Numerous methods that function as pre-screens can be applied to this aim, including SSCP [8], dHPLC [9], FM-CSCE [10], PTT [11] and HR-MCA [12]. Nevertheless, since the cost of sequencing has reduced dramatically it is more appropriate to sequence the full gene now [13]. Direct sequencing of the genomic DNA of the DMD patient identified a nucleotide substitution (c.3604-1G > C) of the last nucleotide of intron 26. Searching in DMD mutation database (www.dmd.nl) revealed that this variant was not described before. This mutation affected the acceptor splice site AG (3′ splice site) of intron 26. Alamut 2.0 Splicing Prediction Module was used in order to evaluate the potential consequences of the mutation on mRNA splicing. According to the online software, the mutation could induce skipping of exon 27. Nevertheless, since skipping of this exon would generate an in-frame mRNA of the *DMD* gene, we further investigated the functional significance of this mutation change. In this view, we analysed the mRNA isolated from peripheral leukocytes of the patient and the control subject. In particular we focused on the region of the *DMD* gene expanding from exon-25 to exon 29. The RT-PCR did not reveal abnormalities in terms of mRNA length, suggesting a correct splicing (Fig. 2a). Nevertheless, direct sequencing of the PCR products evidenced that the first two nucleotides of exon 27 (AG) were absent in the dystrophin mRNA of the DMD patient. The elimination of these two nucleotides from the mRNA shifted the open reading frame (ORF) of exon 27, generating a premature stop codon (UAA) and leading to the absence of dystrophin protein in the skeletal muscle of the DMD patient, as confirmed by immunohistochemistry analysis (Fig. 1). Basing on these evidences, the mechanism proposed is that in the presence of the mutated acceptor splice site of intron 26 (AC), the first two nucleotides of intron 27 are recognized as a new acceptor splice site due to sequence homology and are not included in the mRNA. The elimination of the first two nucleotides AG of the exon 27 generates a STOP codon in exon 27 that does not permit the production of a functional dystrophin protein (c).

## Conclusions

Here we describe a boy who was diagnosed with DMD at 3 years of age on the basis of clinical presentation.and negative dystrophin staining on immunohistochemistry. Our major aim was to identify the mutation responsible for the DMD clinical phenotype and its pathogenic effect on dystrophin translation.

The Best Practice Guidelines for molecular diagnosis of DMD/BMD established that an initial screen detecting the majority of deletions and duplication must be the minimum diagnosis offered. This initial screen can be performed by multiplex PCR technique that screens only 19 exons, the “hot spot”, allowing the identification of about 98 % of all deletions. A more efficient and sensible technique is nevertheless represented by MLPA that allows the quantification of all exons of the *DMD* gene enabling the detection of deletions and duplications at the same time. In our case, we performed a MLPA analysis of genomic DNA that evidenced any exon deletions/duplications. When no exon deletions or duplications are found, and results from muscle biopsy suggest a dystrophinopathy, the clinical diagnosis cannot be confirmed nor excluded (Abbs S Neuromuscular Disorders 2010). In this case, further tests have to be performed in order to identify the pathogenic mutations, like gene sequencing. Interestingly when we sequenced the *DMD* gene a nucleotide substitution (c.3604-1G > C) of the last nucleotide of intron 26 was identified. This mutation affected the acceptor splice site AG (3′ splice site) of intron 26. When a point mutation is within splice consensus sequences, aberrant splicing is likely to happen and maintenance of ORF should be checked by using splice prediction programmes and by analysis of the mRNA. Analysis of the *DMD* gene variant with Alamut 2.0 Splicing Prediction Module predicted that the mutation could induce skipping of exon 27. Since skipping of this exon would generate an in-frame mRNA of the *DMD* gene, we further investigated the pathogenic effect of this mutation on the *DMD* gene. Analysis of the mRNA evidenced that the first two nucleotides of exon 27 (AG) were absent in the dystrophin mRNA of the DMD patient and a premature STOP codon was generated. The mechanism we proposed is that in the presence of the mutated acceptor splice site of intron 26 (AC), the first two nucleotides of intron 27 are recognized as a new acceptor splice site due to sequence homology and are not included in the mRNA. The elimination of nucleotides AG from exon 27 generates a STOP codon in exon 27 that does not permit the production of a functional dystrophin. The speed with which diagnosis is given is relevant and determinant to reduce the anxiety of patients and their family, and to reduce the recurrence of DMD in the family. Mutation is necessary not only to confirm the clinical diagnosis but also enables carrier testing and prenatal diagnosis for family members [14]. In this case report, evaluation of the pathogenic effect of the mutation by mRNA analysis will be useful in the optics of a gene therapy. In case of an antisense oligonucleotides (AON)-based approach, specific oligonucleotides are drawn complementary to the sequence to skip (mutated exons) in order to inhibit its splicing and to correct the ORF of the mutated gene. The resulting protein will be partially functional because it contains both the N- and C-terminal domains that allow a linkage between the extracellular matrix and the cytoskeleton. In alternative, AAV-mediated gene replacement therapy or gene editing approach could be used. Moreover, unrevealing that the mutation in the acceptor splice site of intron 26 (AG → AC) caused a premature stop codon in the *DMD* gene, evidenced that a therapy based on read-through for non-sense mutation can be applied to this patient. Read-through for nonsense mutation is based on administration of small molecules (like gentamicin and Ataluren) that are able to introduce a conformational change in the mRNA structure, thus allowing the ribosomal subunits to substitute a mutation-induced stop codon with a single amino acid [15]. Moreover, on a long term prospective the mutation analysis paves a base for phenotypic-genotypic correlation that throws light in understanding the disorder.

Finally, in dystrophinopathies, not only in DMD, the creation of a good database will facilitate and accelerate the inclusion of patients in clinical trials. This underlines the importance of setting up national patient registries, which can contribute to an international database [16]. At now one the best example of such a database is the TREAT NMD network of excellence (http://www.treat-nmd.eu/home.php).

## Methods

### Immunohistological analysis

Muscle biopsies were collected from the biceps brachialis of the DMD patient and of control subjects. This study was performed according to the guidelines of the Committee on the Use of Human Subjects in Research of the Policlinico Hospital of Milan (Milan, Italy) and the University of Milan. Informed consent was obtained. Biopsies were frozen in liquid nitrogen and muscle slices were cut in order to perform Hematoxylin-Eosin (H&E) staining. Furthermore muscle slices were stained with three dystrophin antibodies (N-terminal Dys-3 antibody, C-terminal Dys-2 antibody and Dys-1 antibody; dilution1/20; NovoCastra, Newcastle-upon-Tyne, United Kingdom).

### Mutation analysis

Blood samples with EDTA were collect from the DMD patients and a control subject. This study was performed according to the guidelines of the Committee on the Use of Human Subjects in Research of the Policlinico Hospital of Milan (Milan, Italy) and the University of Milan. Informed consent was obtained. DNA was isolated from peripheral leukocytes according to standard salting-out protocols. DNA was purified using a commercial kit (E.Z.N.A. Blood DNA Kit II, Peqlab, Erlangen. Germany). Screening for all exons of the dystrophin gene required two independent MLPA kits (SALSA P034 and P035) each with more than 40 individual two-probe sets. MLPA reagents were purchased from MRC Holland (Amsterdam). MLPA experiments were performed following the manufacturer’s protocol. Genome sequencing of the DMD gene was also performed by Primm.

### Reverse transcriptase PCR (RT-PCR)

Dystrophic and control RNA was isolated from peripheral leukocytes using TrizolReagent (Invitrogen, Life Technologies, Carlsbad, California, USA) as described the manufacturer’s protocol and treated with DNAse-RNAse free (Promega, Madison, WI, USA). First-strand cDNA was prepared with the Superscript First Strand-Synthesis System for RT-PCR (Invitrogen Life Technologies), starting from 1 μg of total RNA and oligo (dT)_12–18_ priming. PCR was performed with the following conditions: 94 °C for 5 min, then 30 cycles at 94 °C for 40 s, 60 °C for 40 s, and 72 °C for 1 min. Primers were designed in order to amplify exon 25–29 of DMD transcript and are listed beneath. PCR products were run on 1,5 % agarose gel and specific bands were purified for sequence analysis.

Exon 25: 5′- ATGTGCCAACAGGTCTATGCCAGA -3′:

Exon 29: 5′- TCATCCATGACTCCGCCATCTGTT -3′

### Splicing prediction

The sequence obtained by the analysis of the PCR products referring to DMD sample was analysed using the online software Alamut 2.0 Splicing Prediction Module in order to evaluate if a misreading of the splicing site and skipping of exon 27 happened. (http://www.interactive-biosoftware.com/doc/alamut-visual/2.6/splicing.html).

## Abbreviations

ALT, alanine transaminase; AON, antisense oligonucleotide; AST, aspartate transaminase; BMD, becker muscular dystrophy; CK, creatine kinase; CMV, cytomegalovirus; CSA, cross sectional area; dHPLC, denaturing high performance liquid chromatography; DMD, Duchenne muscular dystrophy; EVC, Ellis van Creveld syndrome; H&E, hematoxylin-eosin; HR-MCA, high resolution melting curve analysis; MLPA, multiplex ligation-dependent probe amplification; MRC, medical research council; NO, nitric oxide; ORF, open reading frame; PCR, polymerase chain reaction; PTT, protein truncation test; SSCP, single-strand conformation polymorphism

## References

[1] Emery AE (1990). Dystrophin function. Lancet.

[2] Bushby K, Finkel R, Birnkrant DJ, Case LE, Clemens PR, Cripe L, Kaul A, Kinnett K, McDonald C, Pandya S (2010). Diagnosis and management of Duchenne muscular dystrophy, part 2: implementation of multidisciplinary care. Lancet Neurol.

[3] Muntoni F, Torelli S, Ferlini A (2003). Dystrophin and mutations: one gene, several proteins, multiple phenotypes. Lancet Neurol.

[4] Den Dunnen JT, Grootscholten PM, Bakker E, Blonden LA, Ginjaar HB, Wapenaar MC, van Paassen HM, van Broeckhoven C, Pearson PL, van Ommen GJ (1989). Topography of the Duchenne muscular dystrophy (DMD) gene: FIGE and cDNA analysis of 194 cases reveals 115 deletions and 13 duplications. Am J Hum Genet.

[5] Beggs AH, Koenig M, Boyce FM, Kunkel LM (1990). Detection of 98 % of DMD/BMD gene deletions by polymerase chain reaction. Hum Genet.

[6] Nobile C, Galvagni F, Marchi J, Roberts R, Vitiello L (1995). Genomic organization of the human dystrophin gene across the major deletion hot spot and the 3′ region. Genomics.

[7] Oudet C, Hanauer A, Clemens P, Caskey T, Mandel JL (1992). Two hot spots of recombination in the DMD gene correlate with the deletion prone regions. Hum Mol Genet.

[8] Mendell JR, Buzin CH, Feng J, Yan J, Serrano C, Sangani DS, Wall C, Prior TW, Sommer SS (2001). Diagnosis of Duchenne dystrophy by enhanced detection of small mutations. Neurology.

[9] Bennett RR, den Dunnen J, O'Brien KF, Darras BT, Kunkel LM (2001). Detection of mutations in the dystrophin gene via automated DHPLC screening and direct sequencing. BMC Genet.

[10] Ashton EJ, Yau SC, Deans ZC, Abbs SJ (2008). Simultaneous mutation scanning for gross deletions, duplications and point mutations in the DMD gene. Eur J Hum Genet.

[11] Roest PA, Roberts RG, Sugino S, van Ommen GJ, den Dunnen JT (1993). Protein truncation test (PTT) for rapid detection of translation-terminating mutations. Hum Mol Genet.

[12] Almomani R, van der Stoep N, Bakker E, den Dunnen JT, Breuning MH, Ginjaar IB (2009). Rapid and cost effective detection of small mutations in the DMD gene by high resolution melting curve analysis. Neuromuscul Disord.

[13] Flanigan KM, von Niederhausern A, Dunn DM, Alder J, Mendell JR, Weiss RB (2003). Rapid direct sequence analysis of the dystrophin gene. Am J Hum Genet.

[14] Abbs S, Bobrow M (1992). Analysis of quantitative PCR for the diagnosis of deletion and duplication carriers in the dystrophin gene. J Med Genet.

[15] Benedetti S, Hoshiya H, Tedesco FS (2013). Repair or replace? Exploiting novel gene and cell therapy strategies for muscular dystrophies. FEBS J.

[16] Aartsma-Rus A, Van Deutekom JC, Fokkema IF, Van Ommen GJ, Den Dunnen JT (2006). Entries in the Leiden Duchenne muscular dystrophy mutation database: an overview of mutation types and paradoxical cases that confirm the reading-frame rule. Muscle Nerve.

